# Lack of PPARγ in Myeloid Cells Confers Resistance to *Listeria monocytogenes* Infection

**DOI:** 10.1371/journal.pone.0037349

**Published:** 2012-05-21

**Authors:** Zeinab Abdullah, Sergej Geiger, Andrea Nino-Castro, Jan P. Böttcher, Eugenia Muraliv, Moritz Gaidt, Frank A. Schildberg, Kati Riethausen, Juliane Flossdorf, Wolfgang Krebs, Trinad Chakraborty, Christian Kurts, Joachim L. Schultze, Percy A. Knolle, Luisa Klotz

**Affiliations:** 1 Institutes of Molecular Medicine and Experimental Immunology, Universität Bonn, Bonn, Germany; 2 Institute for Medical Microbiology, Justus-Liebig-Universität Giessen, Giessen, Germany; 3 Genomics & Immunoregulation, LIMES Institute, Universität Bonn, Bonn, Germany; 4 Clinic for Neurology – Inflammatory Disorders of the Nervous System and Neuro-oncology, Universität Münster, Münster, Germany; National Jewish Health and University of Colorado School of Medicine, United States of America

## Abstract

The peroxisomal proliferator-activated receptor γ (PPARγ) is a nuclear receptor that controls inflammation and immunity. Innate immune defense against bacterial infection appears to be compromised by PPARγ. The relevance of PPARγ in myeloid cells, that organize anti-bacterial immunity, for the outcome of immune responses against intracellular bacteria such as *Listeria monocytogenes in vivo* is unknown. We found that *Listeria monocytogenes* infection of macrophages rapidly led to increased expression of PPARγ. This prompted us to investigate whether PPARγ in myeloid cells influences innate immunity against *Listeria monocytogenes* infection by using transgenic mice with myeloid-cell specific ablation of PPARγ (LysMCre×PPARγ^flox/flox^). Loss of PPARγ in myeloid cells results in enhanced innate immune defense against *Listeria monocytogenes* infection both, *in vitro* and *in vivo*. This increased resistance against infection was characterized by augmented levels of bactericidal factors and inflammatory cytokines: ROS, NO, IFNγ TNF IL-6 and IL-12. Moreover, myeloid cell-specific loss of PPARγ enhanced chemokine and adhesion molecule expression leading to improved recruitment of inflammatory Ly6C^hi^ monocytes to sites of infection. Importantly, increased resistance against *Listeria* infection in the absence of PPARγ was not accompanied by enhanced immunopathology. Our results elucidate a yet unknown regulatory network in myeloid cells that is governed by PPARγ and restrains both listeriocidal activity and recruitment of inflammatory monocytes during *Listeria* infection, which may contribute to bacterial immune escape. Pharmacological interference with PPARγ activity in myeloid cells might represent a novel strategy to overcome intracellular bacterial infection.

## Introduction

The peroxisome proliferator-activated receptor-gamma (PPARγ) is a member of the nuclear hormone-receptor superfamily of ligand-activated transcription factors [1]. It is expressed in different cell types of the immune system, e.g. macrophages/monocytes [2] and lymphocytes [3]. Upon ligand binding, PPARγ heterodimerizes with the retinoid X receptor (RXR) and binds to PPAR response elements located in the promoter region of metabolic target genes [4]. Besides its well-studied role in metabolism and cellular differentiation, PPARγ is a negative regulator of inflammatory gene expression and macrophage activation [5], [6]. PPARγ exerts its anti-inflammatory effects in part by trans-repression, i.e. negative interaction with pro-inflammatory transcription factors like NFκB, or by stabilization of co-repressor complexes such as SMRT or NCoR on promoters of target genes [5]. Among the genes targeted by the inhibitory function of PPARγ are pro-inflammatory cytokines and chemokines but also lineage-determining transcription factors, such as RORγt that promotes differentiation of pro-inflammatory TH17-cells [7]. PPARγ is known to have cross-regulatory function in inhibiting innate immune stimulation elicited through ligand binding to Toll like receptors [8]. Moreover, we and others have demonstrated a negative influence of PPARγ on the immune stimulatory capacity of dendritic cells (DC) [9], [10]. Inhibition of PPARγ in myeloid cells led to induction of systemic inflammation even in the absence of challenge with an infectious agent [11]. There is a large number of endogenous ligands for PPARγ that primarily are derived from arachidonic acid metabolism and are in part induced by immune mediators such as IL-4 and IL-13 [12]. Pharmacological stimulation of PPARγ has been shown to lead to an increased occurrence of bacterial infections in patients [13], suggesting that PPARγ plays a key role in anti-bacterial defense. Also, certain gram-positive bacteria such as Mycobacterium tuberculosis have been shown to increase the expression of PPARγ [14], [15]. However, there is no study on the relevance of PPARγ in myeloid cells for the outcome of bacterial infection, although it is clear that myeloid cells are the key cell population in anti-bacterial defense.

*Listeria monocytogenes* is a facultative intracellular Gram-positive bacterial pathogen. Infection of humans and animals can lead to serious, often fatal disease. In humans, disease is most common among pregnant women, newborns, and immune compromised individuals [16]. Murine listeriosis is used widely as a model to study the immune response against intracellular bacterial infection [17]. Early after infection with *L. monocytogenes*, neutrophils and inflammatory monocytes phagocytose and kill invading bacteria. These phagocytic cells secret TNF and IL-12 [18], [19], [20], that activate NK cells to produce IFNγ, which in turn activates bactericidal effector functions of other phagocytes, such as oxidative burst and production of nitric oxide (NO) [21], [22]. Accordingly, mice lacking these essential inflammatory mediators and mice lacking p47phox oxidase or iNOS, which are deficient in ROS or NO production, all display enhanced susceptibility to *L. monocytogenes* infection [23], [24]. Recruitment of inflammatory monocytes to the site of infection, that is driven by the chemokines MCP1 (CCL2) and MCP3 (CCL7), is an important mechanism supporting development of innate immunity against *L. monocytogenes* infection [25]. Mice lacking CCL2 or CCR2 exhibit diminished inflammatory monocyte recruitment to infection sites resulting in enhanced bacterial growth and overwhelming infection [26].

It has remained unclear whether PPARγ with its potent regulatory activity on innate immune functions in myeloid cells plays a regulatory role during infection with *L. monocytogenes*. Here we address this question and provide clear evidence that PPARγ restricts innate immunity in myeloid cells against *Listeria* infection. As infection with *L. monocytogenes* leads to increased expression of PPARγ our results reveal a so far unrecognized regulatory network in myeloid cells that may be abused by *L. monocytogenes* to counter innate immunity.

## Materials and Methods

### Mice

Wild-type C57BL6/J and PPARγ^flox/flox^ mice [27] were purchased from Charles River, LysM-Cre, IFNγ^−/−^, TNF^−/−^ and CCR2^−/−^ transgenic mice in C57BL6/J background were previously described [28], [29], [30], [31]. All animal studies were approved by local authorities. Animals were bred and kept under specific pathogen-free conditions and used at 8–12 weeks of age in accordance with local animal experimentation guidelines. LysM-specific PPARγ knockout mice (LysM-PPARγ^KO^ mice) were generated by crossing PPARγ^flox/flox^ mice with LysM-Cre^+/−^ transgenic mice expressing Cre-recombinase under control of the LysM promoter. These mice display no alterations in immune cell frequencies.

### Infection of mice and cells

If not indicated otherwise, mice were infected intraperitonealy with 2×10^4^ CFU of wild type *Listeria monocytogenes* (EGDe strain). Cells were infected in antibiotic free medium with *Listeria monocytogenes* at a MOI of 10 for one hour followed by one step wash with PBS and were further cultured in medium containing 50 µg/ml Gentamycin.

### Uptake of fluorochrome labeled Listeria

Murine BMDM or PEC (10^6^ cells) were infected with FITC-labeled listeria (MOI 5 or 10) 1 ml medium. After 30 minutes of incubation, cells were washed with PBS followed by passage through a 30% sucrose layer to get rid of extracellular bacteria. Uptake efficiency was assessed by flow cytometry following staining with anti-Listeria antibody to discriminate between intracellular and extracellular *Listeria*. To determine the infection efficiency of the infected BMDM and PEC, cell lysates were plated on BHI plated and incubated over night at 37°C and CFU/cell was measured. FITC-labeled bacteria were obtained by incubation of wild type *Listeria monocytogenes* for 30 min at 37°C in PBS containing 5 µM FITC (5,6)-fluorescein isothiocyanate mixed isomer (Thermo Scientific)) at a density of 1×10^9^ CFU/ml followed by three times wash with PBS.

### Cells and cell culture

Peritoneal exudates cells (PEC) are collected from the peritoneal cavity of mice by peritoneal lavage using PBS. To achieve macrophage-rich exudates, mice were injected intraperitoneally with 1 ml of 3% thioglycolate and PEC were collected 72 hrs later. Cells were then let to adhere for 1 hr and non- adherent cells were collected. Bone marrow derived macrophages from wild-type or mutant mice were generated by collecting cells from mice tibiae and femurs and culture them in RPMI 1640 medium supplemented with FCS, Glutamate, βME and 30% L929 cells conditioned medium for 7 days. Cells were cultured and infected as previously described [32]. Importantly, for all experiments BMDM were cultured in antibiotic-free medium prior to infection or adoptive transfer studies to eliminate remaining antibiotics that may interfere with bacterial growth. The purity of BMDM generated by the protocol used in this study was routinely >95% as shown by a representative analysis of CD11b and F4/80 expression (**Fig. S1**).

### Transfer of BMDM from LysM-PPARγ^WT^ and LysM-PPARγ^KO^

BMDM were generated as above from LysM-PPARγ^WT^ and LysM-PPARγ^KO^. On days 6, medium was replaced with fresh medium lacking antibiotics. On day 7 of differentiation, 5×10^6^ cells were injected into the peritoneal cavity of IFNγ−/−, TNF−/− or CCR2−/− mice. Two hours later mice were infected with 2×10^4^ CFU/ml of *Listeria monocytogenes*.

### Immunohistochemistry and immunofluorescence microscopy

Murine BMDM (5×10^5^) were seeded on glass coverslips in 24-well plates overnight in antibiotic free medium. Cells were then infected with *L. monocytogenes* at MOI of 10 for 30 min. Cells were then washed with pre-warmed medium and culture further in medium containing 50 µg/ml Gentamycin (Sigma). At indicated time points after infection, cells were washed three times with PBS and were fixed with 4% paraformaldehyde, permeablized with 0.1% Saponin. Cells were incubated with anti-PPARγ in PBS containing 10% FCS and 0.1% Saponin for 1 h. After three washes with PBS, cells were incubated for 1 h at room temperature with FITC–labeled secondary antibody. After that, cells were washed three times with PBS and mounted for fluorescence microscopy. Cells were viewed with a 60 times oil objective lens. Microscopy analysis was performed with an Olympus FluoView IX71Microscope (Olympus).

### Cell culture and infection prior gene expression profiling

Human macrophages were generated from peripheral blood mononuclear cells (PBMC) obtained by Pancoll (PAN-Biotech, Aidenbach, Germany) density centrifugation from buffy coats of healthy donors. CD14^+^ monocytes were then isolated from the PBMC using CD14-specific MACS beads (Miltenyi Biotec) according to the manufacturers protocol (routinely >95% purity). CD14^+^ monocytes were cultured in 6-well plates in RPMI1640 medium containing 10% FCS and differentiated into immature macrophages using GM-CSF (500 U/ml; Immunotools, Friesoythe, Germany) for 72 hours. Only cultures with >95% CD14^+^ CD68^+^ macrophages purity were used for further analysis. Cells were infected with wild type *Listeria monocytogenes* (EGDe strain) using a MOI of 10. 24 hours after infection cells were washed twice and lysed in Trizol (Invitrogen Life Technologies, USA) prior RNA isolation. Only cultures with a cell viability of >80% were used for further analysis.

### Microarray procedure

RNA sample amplification, labeling and hybridization on Illumina HT12 Sentrix BeadChips V4 were performed according to the manufacturer's instructions using an Illumina HiScan SQ. (**Table S1**) summarizes the performed microarray experiments.

### Bioinformatics Analysis

Data analyses were performed using Illumina Genome Studio and Partek Genomics Suite (PGS). Datasets were generated in Illumina and exported to PGS following the manufacturer's instruction. In PGS the quantile method was used for data normalization. Differentially expressed genes were determined using ANOVA. To identify PPARγ target genes regulated during infection of macrophages with *Listeria* a publically available dataset (GSE21314) assessing genome-wide PPARγ binding to DNA by ChIP-Seq was downloaded from GEO. A table of genes most closely located to PPARγ binding sites in macrophages was generated (**Table S2**). The analysis was done with the ChIP-Seq data analysis tool HOMER (Hypergeometric Optimization of Motif EnRichment). This list of genes was used to filter genes differentially regulated in macrophages infected with *Listeria* in contrast to non-infected macrophages (**Table S3**). For visualization of the most differentially expressed PPARγ target genes (n = 80) hierarchical clustering was performed using PGS and plotted as a heatmap. The complete dataset is available at GEO (GSE34103).

### Immunoblot analysis

PPARγ expression was detected by immunoblot using the polyclonal anti-PPARγ (Santa Cruz (H-100)) and anti β-tubulin polyclonal antibody (Licor Bioscience) as previously described [33].

### Serum alanine aminotransferase (ALT) determination

Serum ALT was analyzed from whole blood using ALT strips from Roche according to the manufacturers instructions. Measurement was performed in a Reflovet machine from SCIL animal care.

### Flow cytometry

We used the following fluorochrome-labeled monoclonal antibodies from e-Bioscience: Anti-CD11b-PerCp-cy5.5 (M1/70), F4/80-FITC (BM8), Ly6C-APC (HK1.4), CD11b-Pacific Blue (M1/70) and CD3-PE (G4.18) and the live/dead-APC-Cy7 (near-red) cell staining from Invitrogen. To determine the absolute cell numbers fixed numbers of CaliBRITE**®** Beads (BD) were added to each sample before analysis as internal reference. Analysis, was performed on a FACSCanto (BD) and analyzed data with Flow-Jo**®** software (Tree Star). For flow cytometric analysis of intrahepatic leukocytes, livers were perfused with 20 mL 400 µg/ml collagenase type-IV (Sigma) in Hank's balanced salt solution (HBSS), minced with scissors and subsequently digested for 15 minutes with 400 µg/ml collagenase type-IV in HBSS at 37°C. Digested extracts were pressed through 70-µm cell strainers to gain single-cell suspensions. Liver single cell suspension was subjected to density gradient centrifugation (25% vs 50% Percoll**®** (GE Healthcare)) at 2000 rpm for 20 minutes at 25°C. Leukocytes were collected from the interphase after centrifugation, washed twice with HBSS containing 2% bovine serum albumin and subjected to antibody staining for FACS analysis.

### Determination of cytokines, NO (Nitrite) and ROS

IFNγ, TNF, IL-6, IL-12 were determined in supernatants of cell culture or sera of mice by ELISA using purified and biotinylated antibodies to IFNγ, TNF, IL-6 and IL-12 (e-Bioscience). Nitrite concentrations were determined in the supernatant of cells 24 hrs prior to infection using the Griess Reagent Kit (Invitrogen) according to the manufacturer's protocol. Reactive oxygen species (ROS) production was determined using OxyBURST® Green H2DCFDA (Life Technologies) according to the manufacturer's protocol.

### Quantitative real-time PCR analysis

RNA from macrophages was extracted using the RNeasy mini kit (QIAGEN). Reverse transcription of RNA into cDNA was performed using SuperScript III (Invitrogen). Quantitative Real Time PCR (TaqMann) for PPARγ, IFNγ, TNF, IL-6, IL-12, MCP-1, MCP3, CCR2 and GAPDH expression were performed using pre-designed primers and probes (Gen Expression Assay) from Applied Biosystems on an ABiPrism 7900 HT cycler (Applied Biosystems) according to the manufacturer's instructions. All gene expression data are presented as relative expression to GAPDH.

### Ethics statement

The animal experiments within this manuscript were performed according to the guidelines for animal care enforced by the EU (EU Directive 86/609/EEC) and the state Northrhein Westphalia. The study protocol was approved by the local authorities of the state Northrhein Westphalia (8.87-50.10.31.09.033). Ethics approval for obtaining peripheral blood from healthy volunteers was given by the local ethics committee at the University Hospital Bonn and blood was only drawn from healthy donors after written consent was obtained.

### Statistical analysis

All statistical analysis was performed by Student's *t* test. Significant values are indicated as follows: *, *p*<0.05; **, *p*<0.01; and ***, *p*<0.001.

## Results

### Enhanced expression of PPARγ following *Listeria* infection in macrophages

**Figure 1:**
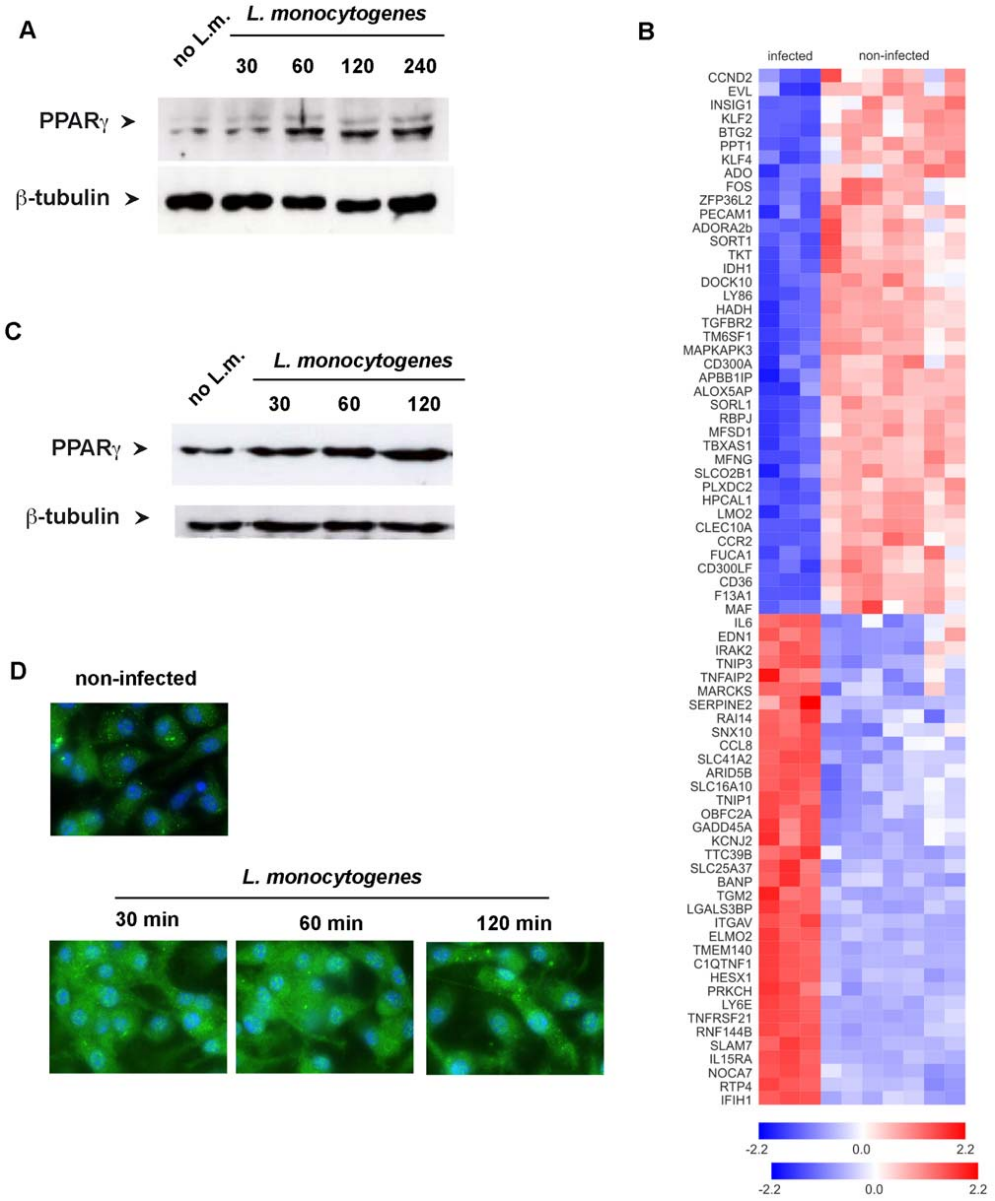
PPARγ expression is induced in bone-marrow derived monocytes after infection with *Listeria monocytogenes*. (A) Time course of PPARγ expression in human monocytes following *Listeria monocytogenes* infection detected by Western blot. (B) PPARγ target genes (n = 80) with the most significant differential expression in human macrophages post infection. Gene expression differences (log scale) are visualized as a heat map following hierarchical clustering of rows and columns (red = increased expression). (C,D) Time course of PPARγ expression in bone marrow derived macrophages (BMDM) post infection detected by Western blot or immunohistochemistry. (blue = DAPI staining the cell nucleus and *Listeria* DNA; green = PPARγ).

As PPARγ is known to be up-regulated during infection of macrophages with *Mycobacteria spp.* or epithelial cells with *Salmonella spp.* [14], [33], we wondered whether infection of macrophages with *Listeria monocytogenes* would also increase PPARγ expression. We observed a rapid and pronounced increase in the expression of PPARγ within 30 minutes after infection, as shown by immunoblot and immunofluorescence analysis of infected human monocytes (**Fig. 1A**). To address the question whether such increase in PPARγ levels were accompanied by transcriptional changes indicative of PPARγ activation, we analyzed the entire transcriptome of *Listeria*-infected human monocytes at a later time point (24 hrs p.i.) compared to non-infected monocytes. We mapped target genes of PPARγ in monocytes recently published as ChIP-seqencing data [34] onto the transcriptome of macrophages infected with *L. monocytogenes* for a longer time period. Of the 891 genes with significant increase in gene expression (FC>2, p<0.05) after infection, 125 genes were PPARγ target genes (**Table S2**). Of the 883 significantly suppressed genes after *L. monocytogenes* infection 100 genes were previously identified to be PPARγ targets. The most differentially expressed genes are shown as a heatmap after hierarchical clustering (**Fig. 1B**). We confirmed these findings for bone marrow derived murine macrophages (BMDM). Already 30 minutes after infection with L. monocytogenes we observed an increase in PPARγ expression that was detected by Western Blot and immunohistochemistry (**Fig. 1C,D** **and suppl.** **Fig. 2D**). Taken together, these results strongly suggested that *Listeria* infection not only led to increased expression of PPARγ but also increased activity as transcriptional regulator.

**Figure 2:**
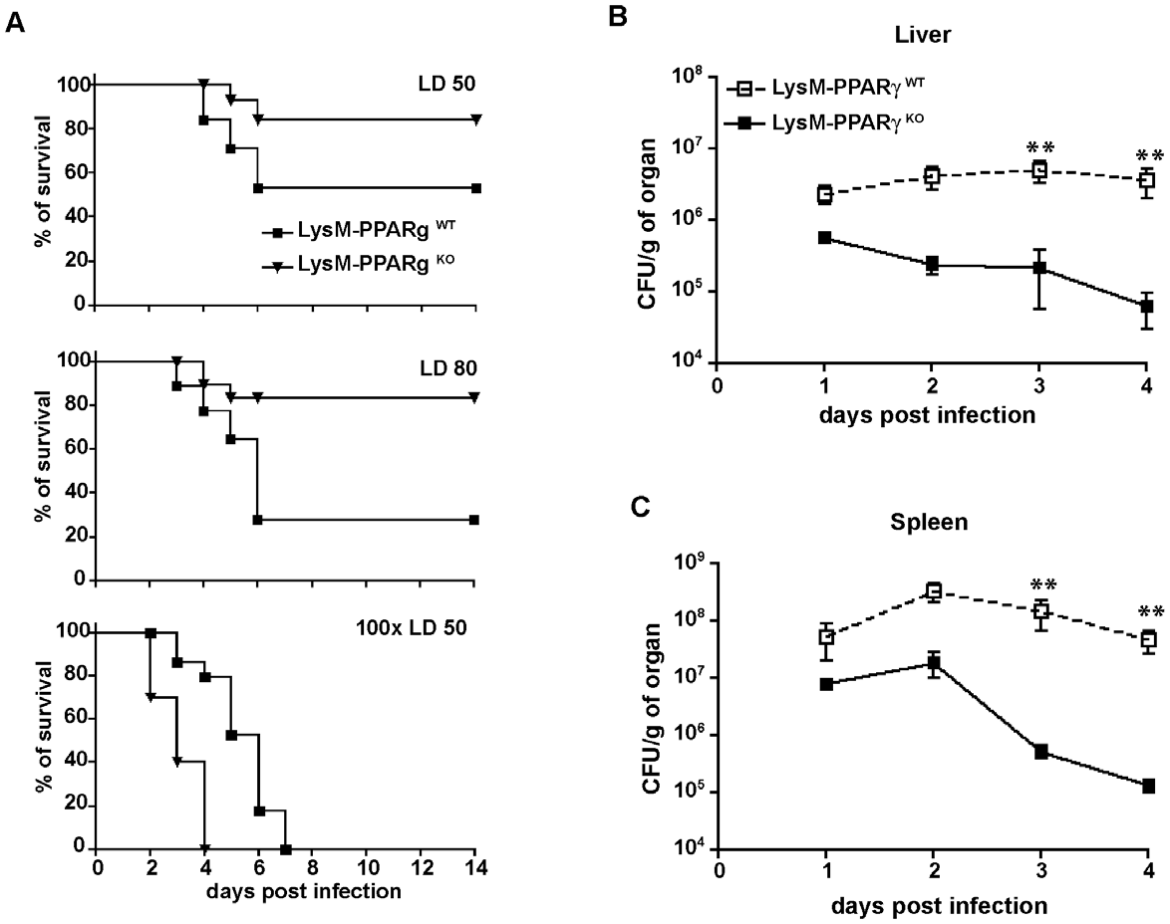
Enhanced resistance of LysM- PPARγ^KO^ mice to lethal infection with *Listeria monocytogenes*. (A) Survival of LysM- PPARγ^KO^ mice and LysM- PPARγ^WT^ littermates after intraperitoneal infection with 2×10^4^ (LD 50) 5×10^5^ (LD 80), 2×10^6^ (100× LD50) CFU of *Listeria*. (n = 10, one representative out of three experiments is shown ). LD 50: p = 0.04; LD 80; 0.01; 100× LD50: not significant difference. (B, C) Colony forming units of *Listeria monocytogenes* in liver (B) and spleen (C) of LysM- PPARγ^WT^ or LysM- PPARγ^KO^ mice infected i.p. with 2×10^4^ CFU. (n = 9); significance ** p = 0.005.

### Myeloid cell-specific PPARγ-ablation augments survival of mice after *L. monocytogenes* infection

Given the early increase of expression of the anti-inflammatory factor PPARγ as well as the differential expression of important PPARγ target genes in *Listeria*-infected macrophages, we next characterized the functional role of PPARγ in myeloid cells in host defense against infection with *L. monocytogenes*. To this end, we generated a transgenic mouse with a myeloid cell-specific knockout of PPARγ by crossing LysM-Cre mice with mice carrying loxP sites within the PPARγ gene (LysMCre×PPARγ^fl/fl^ = LysM- PPARγ^KO^) (**Fig. S2A–C**). The LD_50_ for *Listeria* infection in wildtype littermates (LysM- PPARγ^WT^) was identical to that for wildtype C57Bl/6 mice (2×10^4^ CFU, data not shown). LysM- PPARγ^WT^ and LysM- PPARγ^KO^ mice were challenged with this dose of *L. monocytogenes* and survival was determined. LysM- PPARγ^KO^ mice showed an improved survival compared to wildtype littermates (**Fig. 2A**), indicating that absence of PPARγ in myeloid cells enhances innate immune defense against *Listeria* infection. Almost 80% of LysM- PPARγ^KO^ mice even resisted infection with 20-fold higher numbers of bacteria, a dose where all wildtype littermates succumbed to infection (**Fig. 2A**). However, when infected with very high numbers of bacteria (200 fold LD_50_), LysM- PPARγ^KO^ mice succumbed faster than their wildtype littermates (**Fig. 2A**), revealing a limitation of the gain of protective myeloid cell function upon ablation of PPARγ.

Next, we determined whether the improved resistance to *L. monocytogenes* infection in LysM- PPARγ^KO^ mice was associated with an improved clearance of infecting bacteria. Time kinetic analysis showed already at d1 after infection (2×10^4^ CFU), that LysM- PPARγ^KO^ mice better controlled bacterial infection than LysM- PPARγ^WT^ (**Fig. 2B and C**). In LysM- PPARγ^WT^ mice, little if any decline in bacterial numbers was observed until d4 p.i. In contrast, in LysM- PPARγ^KO^ mice the numbers of *L. monocytogenes* in liver and spleen decreased over the entire time period investigated. At d3 p.i., the bacterial burden in the spleen of LysM- PPARγ^KO^ mice was 100 fold lower compared to LysM- PPARγ^WT^ mice (**Fig. 2C**). Interestingly, increased clearance of bacteria was not accompanied by an exaggerated immune reponse against infected tissue, because only a mild elevation of serum ALT levels was observed in LysM- PPARγ^KO^ mice infected with *L. monocytogenes* (LD50 and LD80) (**Fig. S3**). Taken together, these results suggest that PPARγ in myeloid cells interferes with innate immune responses and bacterial clearance during the early phase of the immune response against intracellular infection with *L. monocytogenes*.

### Loss of PPARγ in myeloid cells enhances bactericidal activity and expression of pro-inflammatory mediators after *L. monocytogenes* infection *in vitro*

**Figure 3:**
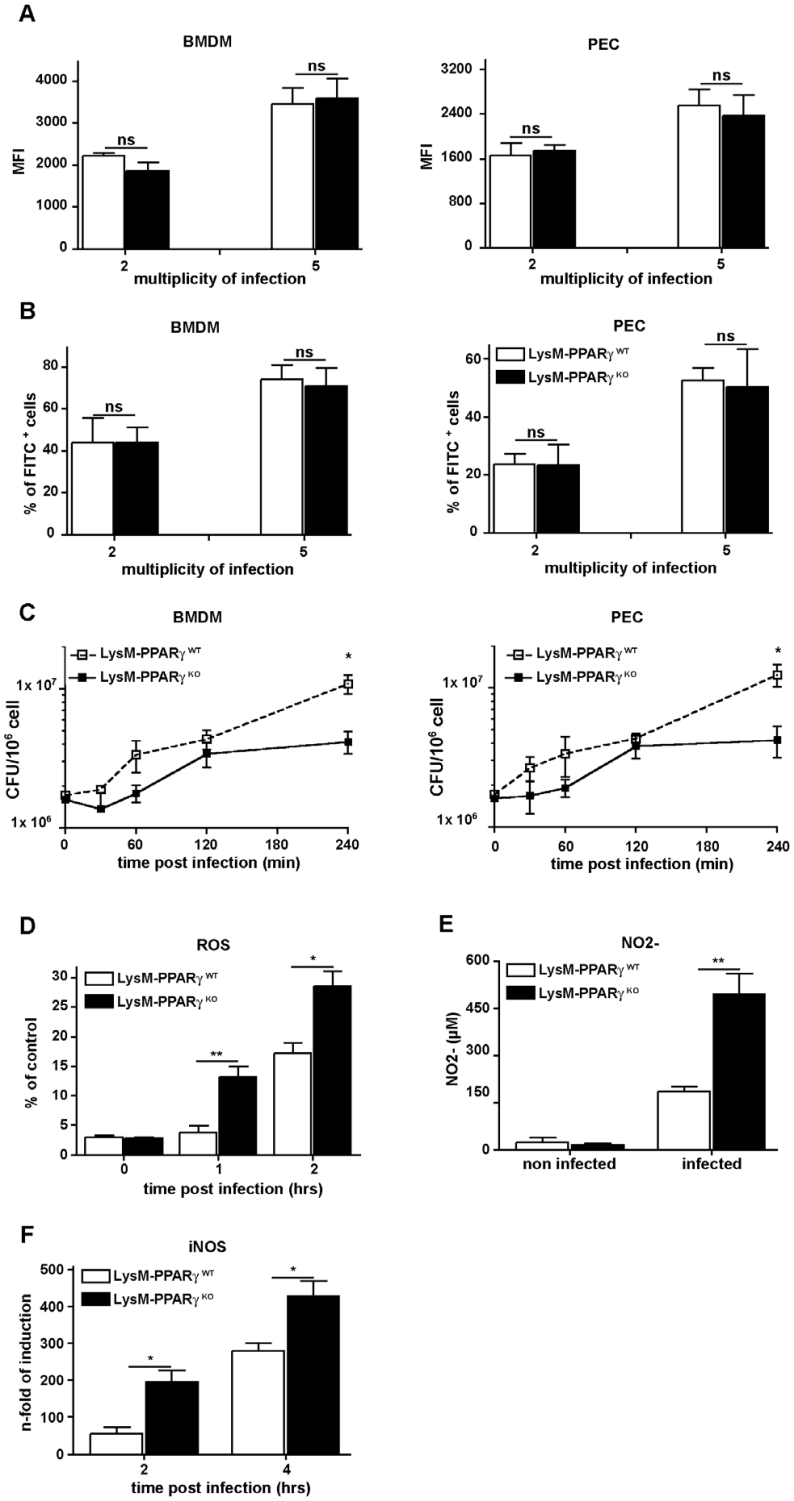
Similar phagocytic capacity in myeloid cells from LysM- PPARγ^WT^ and LysM- PPARγ^KO^ mice. Bone marrow derived macrophages (BMDM) and peritoneal exudates cells (PEC) from LysM- PPARγ^WT^ and LysM- PPARγ^KO^ mice were infected with FITC-labelled *Listeria* at MOI 2 or MOI 5). (A) Mean fluorescence intensity (MFI) of FITC positive cells was assessed by flow cytometry. and (B) Percentage of *Listeria* containing macrophages (FITC positive). (C) BMDM or PEC from LysM- PPARγ^WT^ and LysM- PPARγ^KO^ mice were infected with *Listeria* (MOI 10) and intracellular *Listeria* growth was determined as CFU/ml in the lysates of infected cells. The *p* values for titres of WT vs. KO at 4 hrs p.i. were <0.05 and <0.01 for BMDM and PEC, respectively. (D–F) BMDM from LysM- PPARγ^WT^ and LysM- PPARγ^KO^ mice were infected with *Listeria* (MOI of 10). The capacity to produce reactive oxygen species (ROS) in *Listeria* infected macrophages was determined by the OxyBURST reagent (D). At 24 hrs p.i. the concentration of NO2- as surrogate marker for the production of reactive nitrogen intermediates was determined with Griess reagent (E). Expression of iNOS was analyzed by quantitative RT-PCR (F).

As we observed improved bacterial clearance in LysM- PPARγ^KO^ mice we next investigated the influence of PPARγ on molecular mechanisms known to be critical for control of bacterial infection, such as phagocytosis and generation of listeriocidal mediators like reactive NO and ROS [16], [21]. We did not detect any differences in the extent of phagocytic uptake or the percentage of phagocytic macrophages isolated from bone marrow or peritoneal cavity of either LysM- PPARγ^KO^ or LysM- PPARγ^WT^ mice (**Fig. 3A and B**). Similarly, no difference in phagocytic uptake of fluorescently labeled *L. monocytogenes* was observed (**Fig. 3A and B**), thus excluding that increased phagocytosis by PPARγ^KO^ macrophages was responsible for improved bacterial clearance. Instead, PPARγ^KO^ macrophages more efficiently controlled the growth of intracellular *L. monocytogenes* than PPARγ^WT^ macrophages (**Fig. 3C**). Along this line, we observed that macrophages from LysM- PPARγ^KO^ produced significantly higher level of the listeriocidal mediators ROS and NO2- after infection with *L. monocytogenes* compared to macrophages from LysM- PPARγ^WT^ (**Fig. 3D and E**). As ROS production was more pronounced in PPARγ-deficient macrophages already early during bacterial infection (**Fig. 3D**) this may explain the improved subsequent early control of intracellular bacterial growth. These results were corroborated by the observation that iNOS expression was also enhanced at early time points in *Listeria*-infected PPARγ-deficient macrophages (**Fig. 3F**), findings that are in line with reports that PPARγ controls expression of pro-inflammatory and bactericidal mediators by interfering with NF-κB signaling **[4]**, [6], [35****].

### Absence of PPARγ in myeloid cells augments production of pro-inflammatory mediators upon *L. monocytogenes* infection

**Figure 4:**
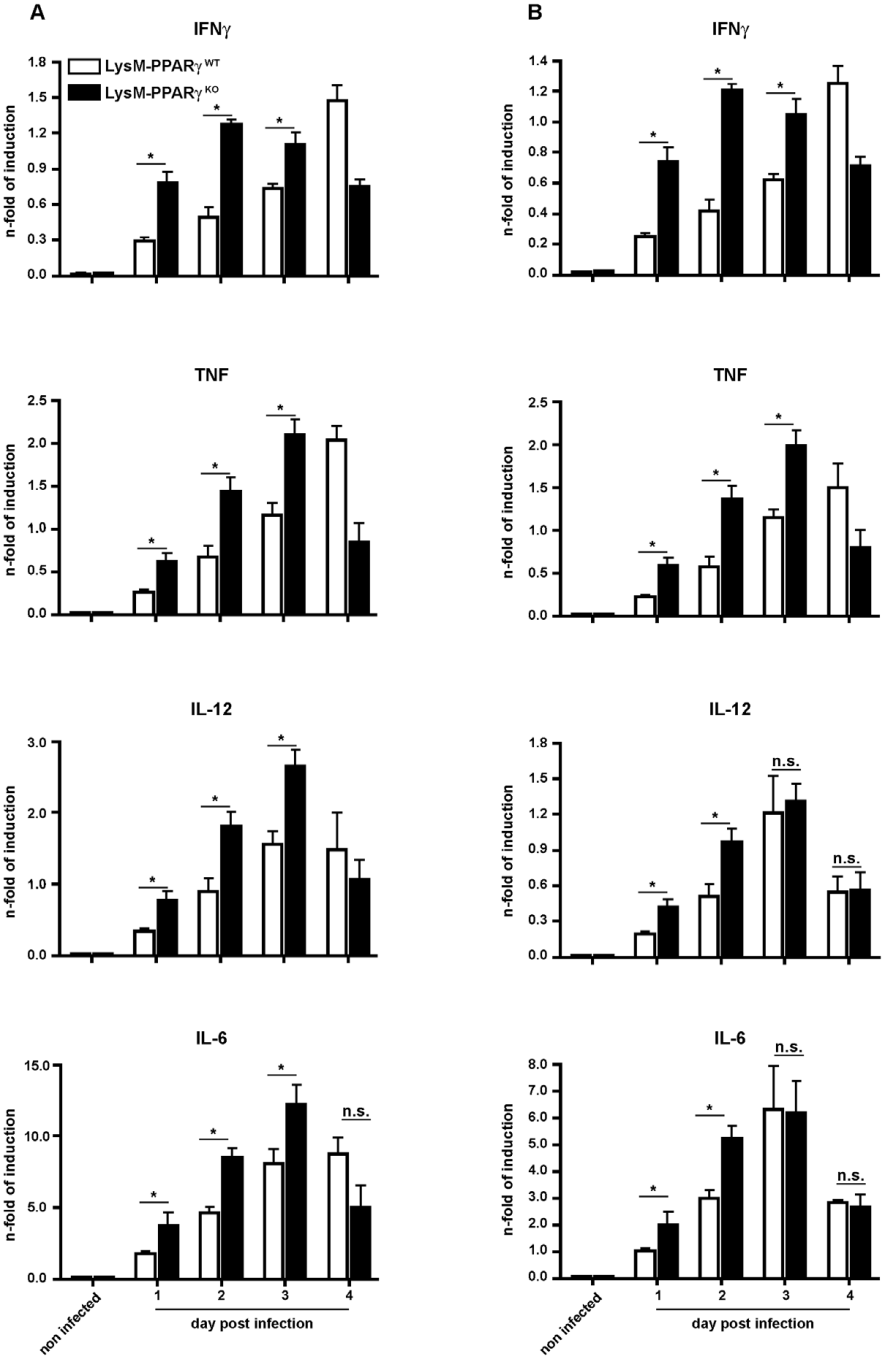
Enhanced expression of inflammatory cytokines in LysM-PPARγ^KO^ mice during infection with *Listeria in vivo*. (A and B) LysM- PPARγ^WT^ and LysM- PPARγ^KO^ mice were infected i.p. with 2×10^4^ CFU of *Listeria*. At indicated time points, expression of IFNγ, TNF, IL-1β, IL-6 and IL-12 was determined in the liver (A) and spleen (B) of mice (n = 5) by qRT-PCR. The data shown are means ± SE of three independent experiments.

Myeloid cells secrete pro-inflammatory mediators, which are essential for innate immune responses against *Listeria* infection [36], [37]. To further characterize the role of PPARγ in myeloid cells during *L. monocytogenes* infection, we determined expression of relevant pro-inflammatory cytokines after *L. monocytogenes* infection *in vivo*. We observed increased expression of IFNγ, TNF, IL-12 and IL-6 in liver and spleen of infected mice (**Fig. 4A and B**) and cytokine expression was found to be significantly higher in organs of PPARγ^KO^ mice at d1-d3 p.i. when compared to PPARγ^WT^ mice (**Fig. 4A and B**). In contrast, at d4 p.i. there was a pronounced reduction of IFNγ and TNF in PPARγ^KO^ compared to PPARγ^WT^ mice (**Fig. 4A and B**), which can be explained by the rapid clearance of *L. monocytogenes* from LysM- PPARγ^KO^ mice as demonstrated before. The early increased induction of proinflammatory cytokines was also observed in peritoneal macrophages isolated from PPARγ^KO^ mice (**Fig. S4**), thus confirming the control function of PPARγ for these cytokines.

**Figure 5:**
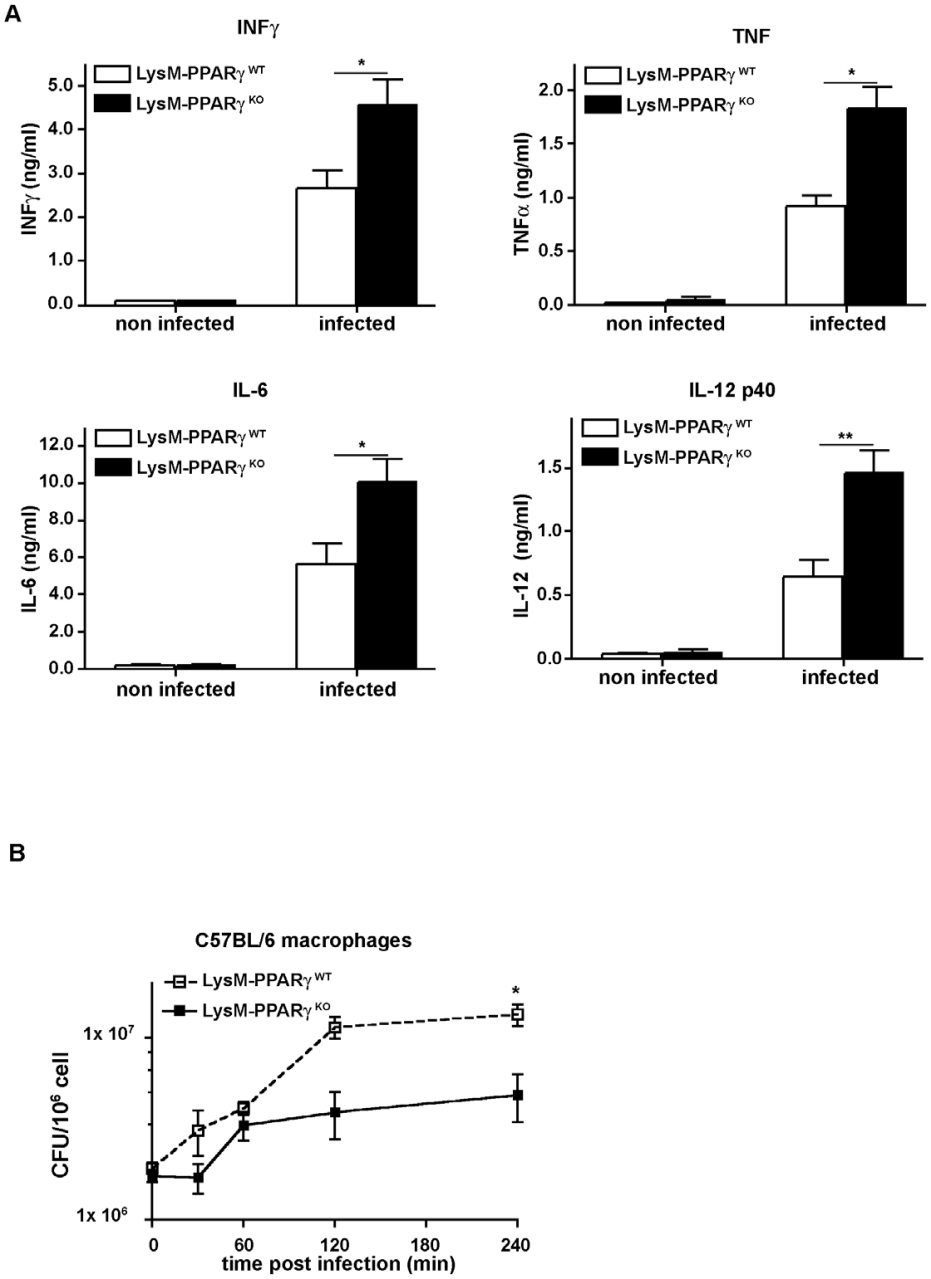
Enhanced effector functions following abelation of PPARγ from myeloid cells. (A) BMDM from LysM- PPARγ^WT^ and LysM- PPARγ^KO^ mice were infected *in vitro* with *L.monocytogenes* at a MOI 10. Expression of IFNγ, TNF, IL-1β, IL-6 and IL-12 was determined 6 hrs post infection in cell culture supernatant by ELISA. The data shown are means ± SE of three independent experiments. (B) Looking at the paracrine effects of PPARγ ablation in myeloid cells, we infected BMDM from wild type C57BL/6 mice with *Listeria* at an MOI of 10 and incubated them with sterile filtered conditioned medium from PPARγ^WT^ or PPARγ^KO^ macrophages infected previously for 18 hrs with *L. monocytogenes*; at the indicated time points intracellular *Listeria* growth was determined as CFU/ml in the lysates of infected cells. Experiments were performed in triplicates. One representative out of four experiments is shown. The *p* value for titers of wt vs. ko at 4 hrs post infection were <0.05.

To provide further evidence for the role of PPARγ in myeloid cells, we analyzed the production of inflammatory cytokines in *L. monocytogenes* infected macrophages derived from PPARγ^KO^ or PPARγ^WT^ mice. Following *L. monocytogenes* infection *in vitro*, macrophages from PPARγ^KO^ mice produced more IFNγ, TNF, IL-6 and IL-12 compared to macrophages from PPARγ^WT^ mice (**Fig. 5A**). These results demonstrate an important cell-intrinsic control function of PPARγ for pro-inflammatory cytokine production in myeloid cells after infection with *L. monocytogenes*. To investigate whether PPARγ-mediated control of macrophage-derived inflammatory cytokines was relevant for bactericidal activity of neighboring cells, macrophages from wildtype C57BL/6 mice were infected with *L. monocytogenes* and cultured in the presence of conditioned medium from *Listeria*-infected peritoneal macrophages isolated from LysM- PPARγ^KO^ or LysM- PPARγ^WT^ mice. While wildtype macrophages incubated in conditioned medium from infected PPARγ^KO^ macrophages were able to restrict the growth of intracellular *Listeria* immediately after infection, macrophages cultured in conditioned medium from wild type macrophages did not rapidly control *Listeria* growth (**Fig. 5B**). These results indicate that ablation of PPARγ in myeloid cells also functioned in a paracrine fashion to increase anti-bacterial immunity in neighboring cells by proinflammatory cytokines.

### Deletion of PPARγ in myeloid cells augments recruitment of inflammatory monocytes to sites of *L. monocytogenes* infection *in vivo*

**Figure 6:**
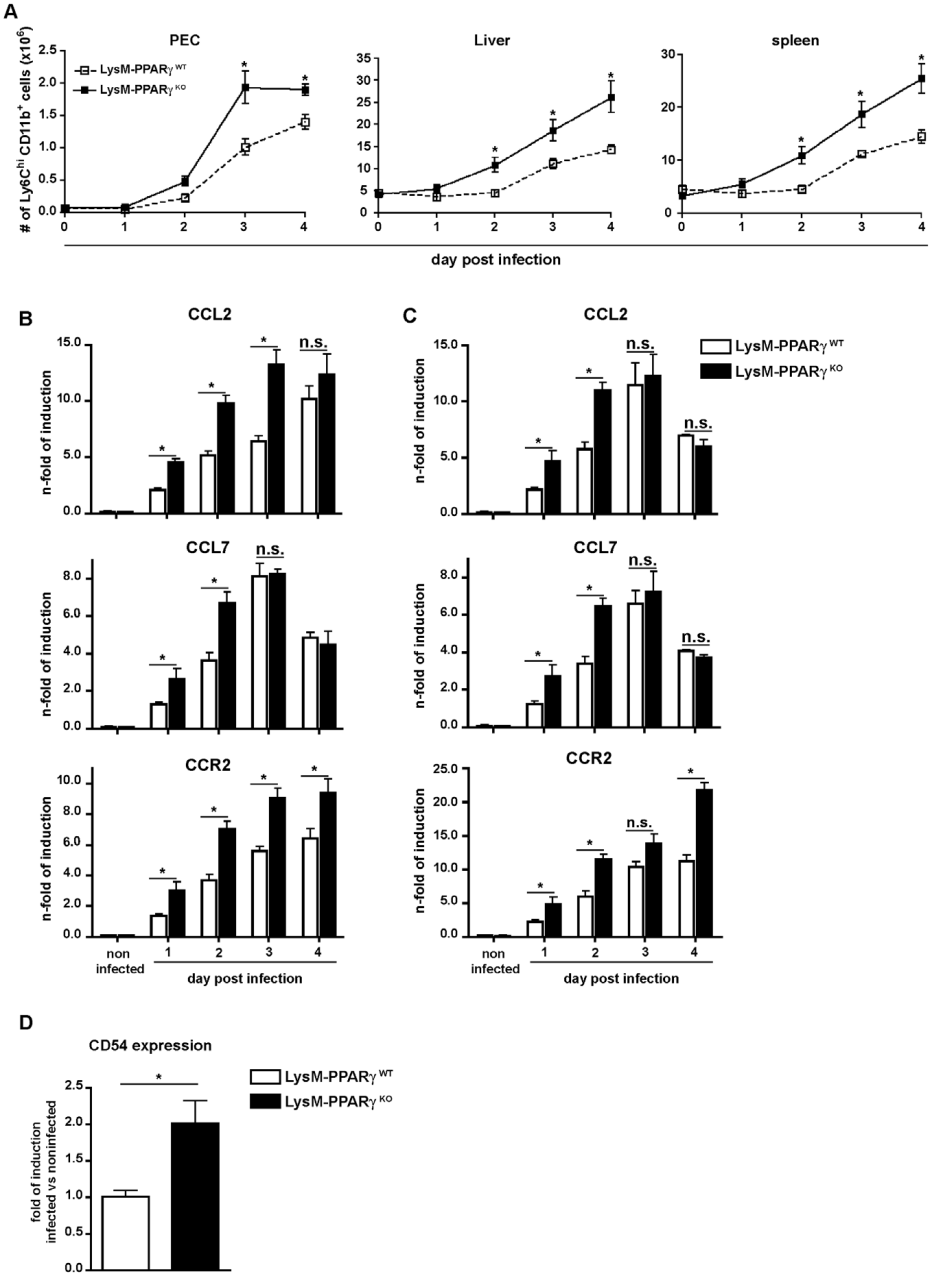
Enhanced recruitment of inflammatory monocytes to the site of infection in LysM-PPARγ^KO^ mice. LysM- PPARγ^WT^ and LysM- PPARγ^KO^ mice were infected i.p. with *Listeria* (2×10^4^ CFU). (A) at indicated time points total numbers of CD11b^+^ Ly6C^high^ monocytes in the peritoneal cavity (PEC), liver and spleen were determined by FACS analysis (B–C) Expression of CCL2, CCL7 and CCR2 in the liver (B) and spleen (C) of infected mice as determined by qRT-PCR. (D) LSEC isolated from wild type C57BL/6 mice were incubated *in vitro* with sterile filtered supernatant peritoneal macrophages from LysM- PPARγ^WT^ and LysM- PPARγ^KO^ mice that were either infected (18 hrs) or left non-infected. The fold increase in expression of CD54 on the surface of LSEC determined by increase in mean fluorescence intensity by flow cytometry in response to contact with supernatant from infected vs non-infected peritoneal macrophages was determined after 24 hrs.

The recruitment of inflammatory monocytes that produce TNF and NO for control of *Listeria* infection has been demonstrated to be crucial for anti-bacterial innate immune defense [38]. Clearly, the recruitment of Ly6C^high^CD11b^+^ inflammatory monocytes to sites of infection was increased from d2 p.i. in LysM- PPARγ^KO^ mice compared to LysM- PPARγ^WT^ mice (**Fig. 6A** **and Fig. S5**). The increased number of inflammatory monocytes in LysM- PPARγ^KO^ mice was not caused by reduced apoptosis as measured by expression of activated Caspase 3/7, bax, bcl2 by Western blotting (**Fig. S6**). As recruitment of these cells requires chemokines such as CCL2, CCL7 [23], [25] and adhesion molecules such as CD54 [39], we investigated the expression of these molecules in LysM- PPARγ^KO^ mice. We detected a similar up-regulation of these genes in lysates from liver (**Fig. 6B**) and spleen (**Fig. 6C**) of LysM- PPARγ^KO^ mice. Consistent with this finding, in myeloid cells isolated from the peritoneal cavity after *L. monocytogenes* infection we found a significant increase in gene expression for CCL2 and CCL7 as well as the corresponding chemokine receptor CCR2, which was most pronounced at d1 and d2 after infection (**Fig. S7**). As entry of inflammatory monocytes into infected liver requires CD54 expression on liver endothelium [39], we exposed primary liver sinusoidal endothelial cells *in vitro* to supernatants of *Listeria*-infected macrophages from PPARγ^KO^ or LysM- PPARγ^WT^ mice. There was a significant induction of CD54 expression on LSEC when exposed to supernatants of *Listeria*-infected but not non-infected macrophages from PPARγ^KO^ mice (**Fig. 6D**), which suggests that macrophages from PPARγ^KO^ mice secrete mediators such as TNF and IFNγ that act on LSEC to upregulate CD54 expression. These results indicate that PPARγ in myeloid cells controls both, chemokine as well as adhesion-molecule driven recruitment of inflammatory monocytes to sites of infection.

### PPARγ ablation renders myeloid cells more competent to control *L. monocytogenes* infection *in vivo*

**Figure 7:**
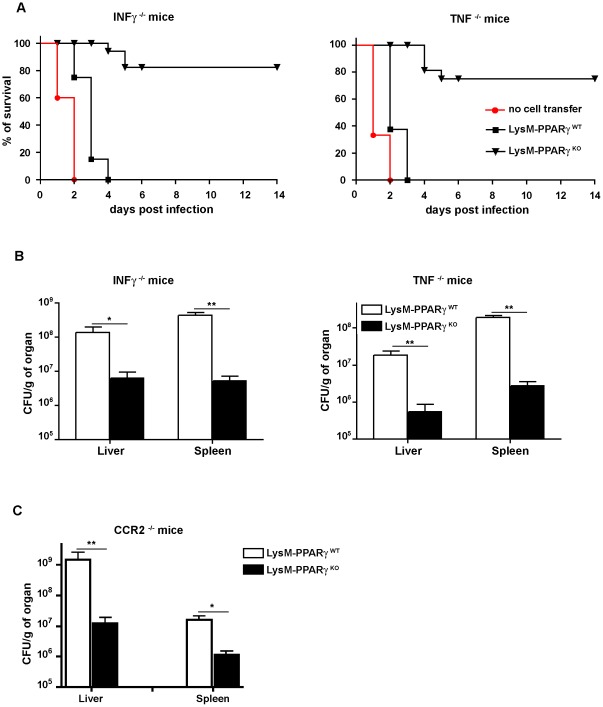
Transfer of PPARγ-deficient macrophages increases the resistance of IFNγ^−/−^, TNF^−/−^ and CCR2^−/−^ mice to *Listeria* infection. 5×10^6^ BMDM from LysM- PPARγ^WT^ or LysM- PPARγ^KO^ mice were adoptively transferred into the peritoneal cavity of IFNγ^−/−^, TNF^−/−^ or CCR2^−/−^ mice and two hours later mice (n = 10) were infected with 2×10^4^ CFU of *Listeria*. (A) Survival of IFNγ^−/−^ and TNF^−/−^ mice after infection with *Listeria*. Knockout mice not receiving any cells by adoptive transfer served as controls. Significance for results in IFNγ^−/−^ and TNF^−/−^ mice were p<0.0001. (B and C) On day 2 after infection, CFU were determined in the homogenates of spleen and liver. One representative out of three independent experiments is shown.

Finally, we elucidated the relevance of PPARγ-mediated regulation in myeloid cells to clear *L. monocytogenes* infection *in vivo*. As the soluble mediators IFNγ and TNF are crucial to control infection of mice deficient for either of these mediators that rapidly succumb to infection [40], [41], we chose to adoptively transfer macrophages from LysM- PPARγ^KO^ or LysM- PPARγ^WT^ into the peritoneal cavity of IFNγ^−/−^ or TNF^−/−^ mice prior to infection of the mice. All knockout mice succumbed on day 2 p.i. and those mice that received wildtype macrophages succumbed on day 3 p.i. (**Fig. 7A**). In contrast, 75–80% of IFNγ^−/−^ or TNF^−/−^ mice that received PPARγ^KO^ macrophages survived until the end of the observation period (**Fig. 7A**). Direct comparison of the bacterial load in organs from these mice at d2 p.i. revealed that only IFNγ^−/−^ or TNF^−/−^ mice receiving PPARγ^KO^ macrophages had lower levels of bacteria in liver and spleen compared to mice receiving PPARγ^WT^ macrophages (**Fig. 7B**). These results clearly demonstrate that ablation of PPARγ renders myeloid cells more competent to control infection *in vivo* presumably through the increased expression of soluble mediators as transfer of these cells restored resistance towards *Listeria* infection in cytokine-deficient animals. To address the question whether the gain of function upon PPARγ ablation in macrophages is already sufficient to improve control of *Listeria* infection in the absence of inflammatory monocytes, we adoptively transferred PPARγ-deficient macrophages into CCR2^−/−^ mice [25]. Under these experimental conditions, we also observed reduced numbers of bacteria in the liver and spleen (**Fig. 7C**), which indicates that the improved function of PPARγ-deficient macrophages alone is already sufficient to control infection. Collectively, we report here the discovery of a so far unknown regulatory network in myeloid cells that is governed by PPARγ and which plays a key role in the control of innate immune responses to *L. monocytogenes* infection.

## Discussion

The immune response against infection with intracellular bacteria is orchestrated by myeloid cells [16], [36], [37]. These cells initiate and amplify innate immune responses leading to immediate reduction of bacteria or infected cells, and also serve as important link for the induction of adaptive immunity to achieve long-lasting protective immunity. Here, we have investigated the role of the potent anti-inflammatory transcription factor PPARγ in regulating the early innate immune response of myeloid cells against infection with *L. monocytogenes*. Our results identify a so far unknown central role of PPARγ in the coordination of listeriocidal functions and recruitment of inflammatory monocytes to the site of infection.

The expression of PPARγ has been shown to be augmented in macrophages after infection with pathogenic bacteria such as *Mycobacterium tuberculosis* and *Salmonella enteridis* [14], [15], [33]. Here, we demonstrate that also the intracellular bacterium *L. monocytogenes* increases PPARγ expression rapidly within 30 minutes after infection of primary murine macrophages. The molecular mechanism underlying increased expression of PPARγ remains unclear and will require further investigation. It is important to note, however, that this induction of a regulatory transcription factor by *Listeria* infection occurs immediately upon infection and before an inflammatory response was mounted. This is clearly different from immunoregulatory mediators such as IL-10, which typically are expressed after inflammation has been induced and serves in many infections to restrict detrimental immunopathology [42]. Although there is evidence that autoactivation may occur if large numbers of PPARγ are present in a cell [43], PPARγ is a ligand-activated receptor raising the question whether increased expression levels were also associated with PPARγ-activation. Gene expression analysis of *Listeria monocytogenes* infected macrophages revealed regulation of PPARγ responsive gene sets indicating that PPARγ has likely undergone ligand-activation after infection. It is possible that other cell populations such as mast cells through production of IL-4 may contribute to increased production of endogenous PPARγ ligands *in vivo* [44]. However, our data demonstrating increased bactericidal function of PPARγ^KO^ compared to PPARγ^WT^ macrophages against *Listeria* infection *in vitro* suggest that there is also induction of endogenous PPARγ ligands in macrophages themselves. It appears likely that *L. monocytogenes* infection induced increased expression of endogenous ligands as we have previously reported that following *Listeria* infection of myeloid cells COX2 expression was induced [45], that leads to generation of prostanoids that can serve as PPARγ ligands.

Given the potent anti-inflammatory effects of PPARγ by transrepression of NF-kB and stabilization of co-repressor complexes on promoters of pro-inflammatory genes [1], such induction of PPARγ in macrophages upon bacterial infection may give rise to escape from innate immune responses acting on various immune effector mechanisms, because PPARγ targets many pro-inflammatory genes [8]. We investigated this question by generating transgenic mice lacking PPARγ in myeloid cells. Indeed, myeloid-cell specific knockout of PPARγ rendered mice more resistant to infection with *L. monocytogenes* supporting the assumption that PPARγ expression can help bacteria to evade the early phases of innate immunity. Lack of PPARγ in myeloid cells resulted in increased expression of essential inflammatory mediators such as TNF, IFNγ and IL-12, which in a paracrine fashion stimulated further IFNγ expression by NK cells (data not shown). Our findings of increased expression in PPARγ-deficient myeloid cells are consistent with earlier reports that pro-inflammatory mediators such as TNF and IFN are target genes of PPARγ [34]. The lack of PPARγ in myeloid cells also increased expression of iNOS and production of the listeriocidal NO, which most likely occurred indirectly through the augmented production of pro-inflammatory mediators, because iNOS does not belong to PPARγ-regulated genes [34].

In addition to increased bactericidal activity, PPARγ ablation in myeloid cells resulted in enhanced recruitment of inflammatory monocytes to the site of infection. We observed increased expression of the key chemokines relevant for inflammatory monocyte recruitment, i.e. CCL2 and CCL7 [46] as well as their receptor CCR2, in the absence of PPARγ in myeloid cells. CCL2 and CCR2 are target genes of PPARγ [34], which again supports our notion that PPARγ regulates anti-bacterial immunity in myeloid cells in a cell-intrinsic fashion. As inflammatory monocytes differentiate into dendritic cell subset, so-called tipDCs, within inflamed tissues [26], it is possible that PPARγ also controls the numbers of tipDCs at the site of infection. This assumption is supported by a recent publication demonstrating that pharmacologic PPARγ activation reduced chemokine-driven recruitment and local proliferation of tipDCs during viral infection [47]. While chemokine-expression is sufficient for the recruitment of CCR2^+^ monocytes into the spleen [46], expression of CD54 (ICAM-1) was shown to be required for the recruitment of these cells to the liver [39]. Here, we show that ablation of PPARγ in myeloid cells also led to release of mediators that subsequently increased expression of CD54 on liver sinusoidal endothelial cells, the cell population that is responsible for recruitment of immune cells from blood passing through the liver [39], [48]. The recruitment of inflammatory monocytes is of key importance in immune defense against bacterial, fungal and parasite infections [38], [49]. Thus, our results demonstrate that PPARγ in myeloid cells has a central role in controlling both, the orchestration of bactericidal activity and immune cell recruitment to the site of infection.

PPARγ has been reported to have anti-inflammatory effects in many immune cell populations. Recently, we have shown that lack of PPARγ in T cells facilitates RORγt-mediated development of pro-inflammatory T_H17_ cells and thereby promotes central nervous system autoimmunity [7]. Activation of PPARγ in dendritic cells impairs their ability to elicit T cell mediated immunity [10]. As augmented expression of PPARγ and increased expression of endogenous ligands generated by the enzyme 12/15-lipoxygenase are observed under inflammatory conditions [44], it is possible that PPARγ plays a role in the prevention of overzealous immunity within inflamed tissues, similar to the expression of co-inhibitory molecules like B7H1 acting on PD1 on T cells to prevent organ immunopathology [50]. In fact, PPARγ in intestinal epithelial cells is essential to maintain absence of inflammation from the gut [33]. Our findings raise the question whether absence of PPARγ from myeloid cells, which improves early anti-bacterial innate immunity, comes at the price of increased immunopathology to infected organs. However, we did not observe any increase in liver injury in mice infected with an LD_50_ of *Listeria monocytogenes* lacking PPARγ in myeloid cells (data not shown), indicating that improved anti-bacterial immunity was not accompanied by increased immunopathology. Even at higher doses of bacteria (LD_80_) we did not observe immunopathology to infected organs such as the liver in LysM- PPARγ^KO^ mice. Only when challenged with very high numbers of bacteria (100× LD_50_) this resistance to infection was broken and mice lacking PPARγ in myeloid cells then died even more rapidly than their wildtype littermates. The balance between pro- and anti-inflammatory signals, such as IL-10, TGFβ and factors released from regulatory T cells, decides about the outcome between immunity and immunopathology to viruses [51], and generation of regulatory macrophages restricts immunity to bacterial infection [52]. Whereas all these regulatory mechanisms act during the late innate or early adaptive phases of the immune response against infectious pathogens, our results reveal a so far unrecognized early control of anti-bacterial innate effector mechanisms through PPARγ activation restricting inflammation, which are not coupled to immunopathology once inactivated. As survival of *L. monocytogenes* in myeloid cells such as dendritic cells is critical for the successful establishment of infection [53], [54], it appears likely that induction of PPARγ by *L. monocytogenes* is a critical step during infection and may serve as early escape from innate immunity.

Taken together our results support the notion that PPARγ represents a valuable target for pharmacologic intervention that when neutralized during bacterial infection would lead to preferential enhancement of immune-mediated clearance of intracellular bacteria such as *L. monocytogenes* without accompanying immunopathology to infected organs like the liver.

## Supporting Information

Figure S1 Purity of bone marrow derived macrophages obtained from LysM- PPARγ^WT^ and LysM- PPARγ^KO^ mice. Cells were generated as described in Materials and Methods and on day 7 cells were stained with αCD11b-PerCp-Cy5.5 and F4/80-FITC.
(TIF)

Figure S2 PPARγ ablation in myeloid cells. (A, B) RT-PCR analysis of mRNA isolated from bone marrow derived macrophages, neutrophil granulocytes, CD11c^+^ splenic cells, CD4^+^ or CD8^+^ T cells and B cells derived from LysM- PPARγ^KO^ (A) or from bone marrow derived macrophages from LysMCrexPPARγ^flox/flox^, LysMCrexPPARγ^WT^, PPARγ^flox/flox^ and C57BL/6 mice or CD8^+^ T cells from LysMCrexPPARγ^flox/flox^ mice. Expression of Cre-recombinase in myeloid cells results in a specific deletion of exons 1 and 2 of the PPARγ gene shown by a truncated 300 bp fragment of PPARγ cDNA, in contrast to the full-length 700 bp wildtype cDNA. For the remainder of the manuscript LysMCrexPPARγ^flox/flox^ are referred to as LysM- PPARγ^KO^ (C) The decrease of PPARγ at the protein level was confirmed by immunoblot in lysates of peritoneal macrophages from LysMCrexPPARγ^flox/flox^, LysMCrexPPARγ^WT^ and PPARγ^flox/flox^ mice. (D) Infecting *L. monocytogenes* were detected by DAPI-staining. For better visibility gray scale conversion of immunofluorescence pictures are shown. Some infecting *Listeria* are indicated by arrow heads.
(TIF)

Figure S3 Determination of serum ALT levels. Alanine aminotransferase levels in serum were determined in mice LysM-PPARγ^WT^ and LysM-PPARγ^KO^ mice following infection with different doses of *L. monocytogenes*.
(TIF)

Figure S4 Enhanced expression of inflammatory cytokies in PPARγ^KO^ macrophages during *Listeria* infection *in vivo*. LysM-PPARγ^WT^ and LysM-PPARγ^KO^ mice were infected with 2×10^4^ CFU of *L. monocytogenes*. At indicated time points peritoneal macrophages were isolated and the expression of IFNγ, TNFα, IL-6 and IL-12 was assessed by qRT-PCR.
(TIF)

Figure S5 Gating strategy and representative flow cytometric data for increase of inflammatory monocytes. LysM-PPARγ^WT^ and LysM-PPARγ^KO^ mice were infected i.p. with *Listeria* (2×10^4^ CFU). (A) Gating strategy for identifying viable inflammatory monocytes. (B) At indicated time points the frequency of CD11b^+^ Ly6C^high^ monocytes within viable cells isolated from the liver were determined by FACS analysis. Quantification of inflammatory monocyte cell numbers was done using fluorochrome-labeled microbeads (CountBright absolute counting beads, life technologies, Invitrogen).
(TIF)

Figure S6 Annexin V staining of inflammatory monocytes from LysM-PPARγ^WT^ and LysM-PPARγ^KO^ mice. Detection of Annexin V levels on the surface of inflammatory monocytes isolated from the liver of Listeria-infected LysM-PPARγ^WT^ and LysM-PPARγ^KO^ mice at the indicated time points. 3 mice per group were analysed, one out of three representative experiments is shown.
(TIF)

Figure S7 Enhanced expression of CCL2 and CCL7 and their receptor CCR2 in PPARγ^KO^ macrophages during *Listeria* infection *in vivo*. LysM-PPARγ^WT^ and LysM-PPARγ^KO^ mice were infected with 2×10^4^ CFU of *Listeria*. At indicated time points peritoneal macrophages were isolated and the expression of CCL2, CCL7 and CCR2 was assessed by qRT-PCR.
(TIF)

Table S1 List of arrays performed.
(DOCX)

Table S2 List of genes most closely located to PPARγ binding sites in macrophages.
(XLSX)

Table S3 List of genes differentially regulated in macrophages infected with *Listeria* in compared to non-infected macrophages.
(XLSX)
