# Supplementary figures and images for: Lack of PPARγ in Myeloid Cells Confers Resistance to Listeria monocytogenes Infection
Source: PLoS One. 2012 May 21;7(5):e37349. doi: 10.1371/journal.pone.0037349 (PMC3357414; doi:10.1371/journal.pone.0037349)

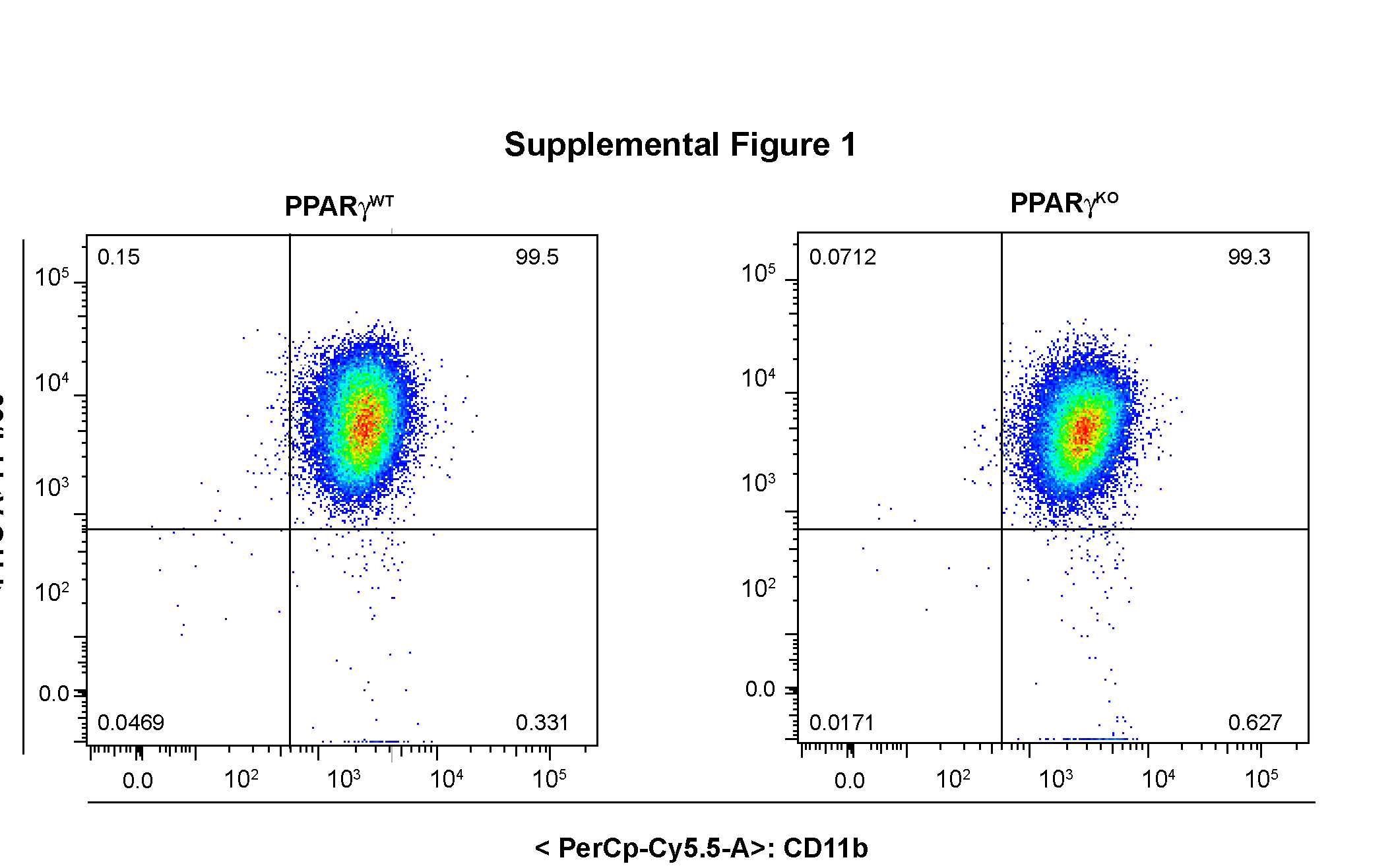

Supplement: Figure S1 — Purity of bone marrow derived macrophages obtained from LysM- PPARγWT and LysM- PPARγKO mice. Cells were generated as described in Materials and Methods and on day 7 cells were stained with αCD11b-PerCp-Cy5.5 and F4/80-FITC. (TIF) [file pone.0037349.s001.tif]

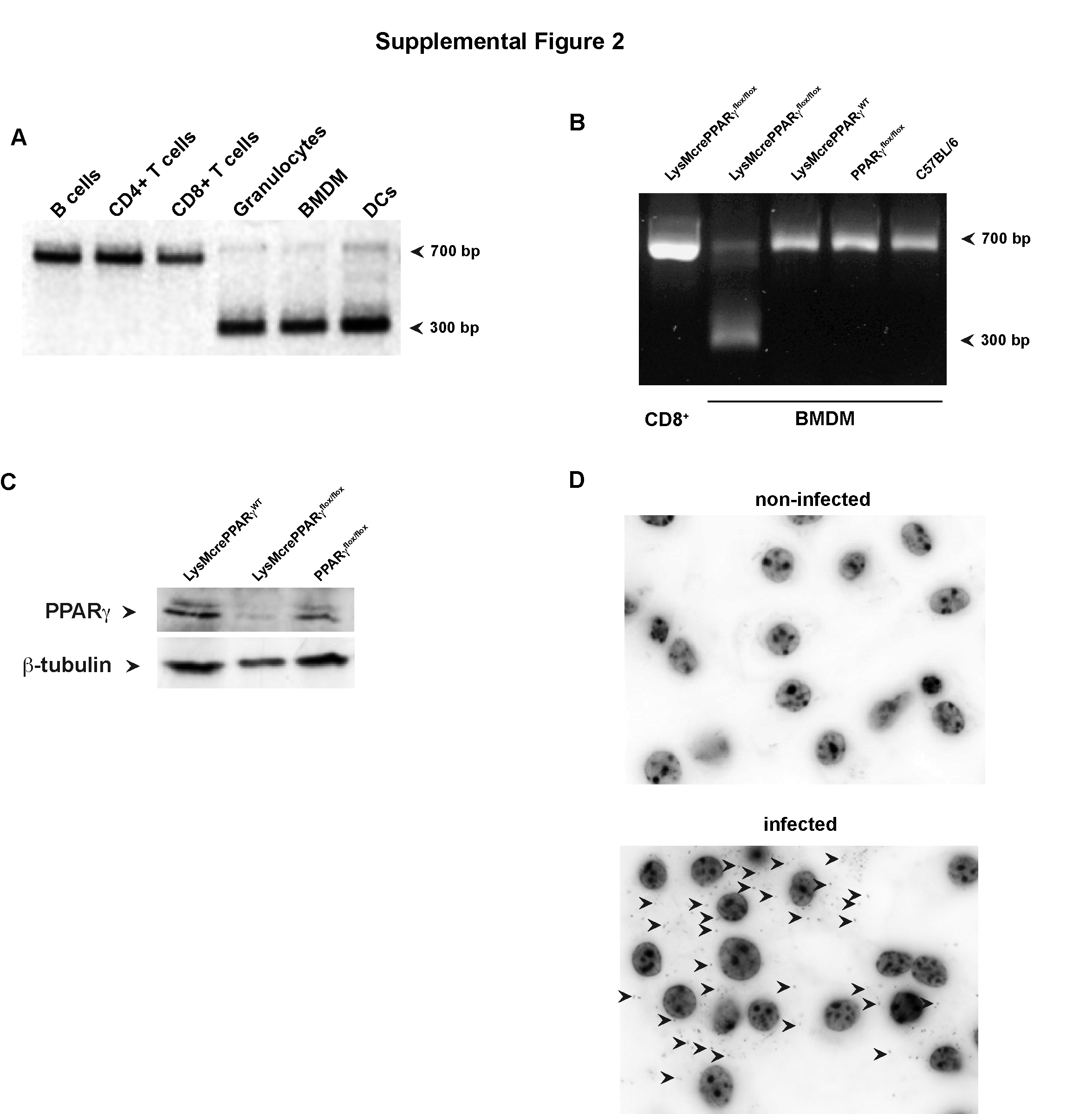

Supplement: Figure S2 — PPARγ ablation in myeloid cells. (A, B) RT-PCR analysis of mRNA isolated from bone marrow derived macrophages, neutrophil granulocytes, CD11c+ splenic cells, CD4+ or CD8+ T cells and B cells derived from LysM- PPARγKO (A) or from bone marrow derived macrophages from LysMCrexPPARγflox/flox, LysMCrexPPARγWT, PPARγflox/flox and C57BL/6 mice or CD8+ T cells from LysMCrexPPARγflox/flox mice. Expression of Cre-recombinase in myeloid cells results in a specific deletion of exons 1 and 2 of the PPARγ gene shown by a truncated 300 bp fragment of PPARγ cDNA, in contrast to the full-length 700 bp wildtype cDNA. For the remainder of the manuscript LysMCrexPPARγflox/flox are referred to as LysM- PPARγKO (C) The decrease of PPARγ at the protein level was confirmed by immunoblot in lysates of peritoneal macrophages from LysMCrexPPARγflox/flox, LysMCrexPPARγWT and PPARγflox/flox mice. (D) Infecting L. monocytogenes were detected by DAPI-staining. For better visibility gray scale conversion of immunofluorescence pictures are shown. Some infecting Listeria are indicated by arrow heads. (TIF) [file pone.0037349.s002.tif]

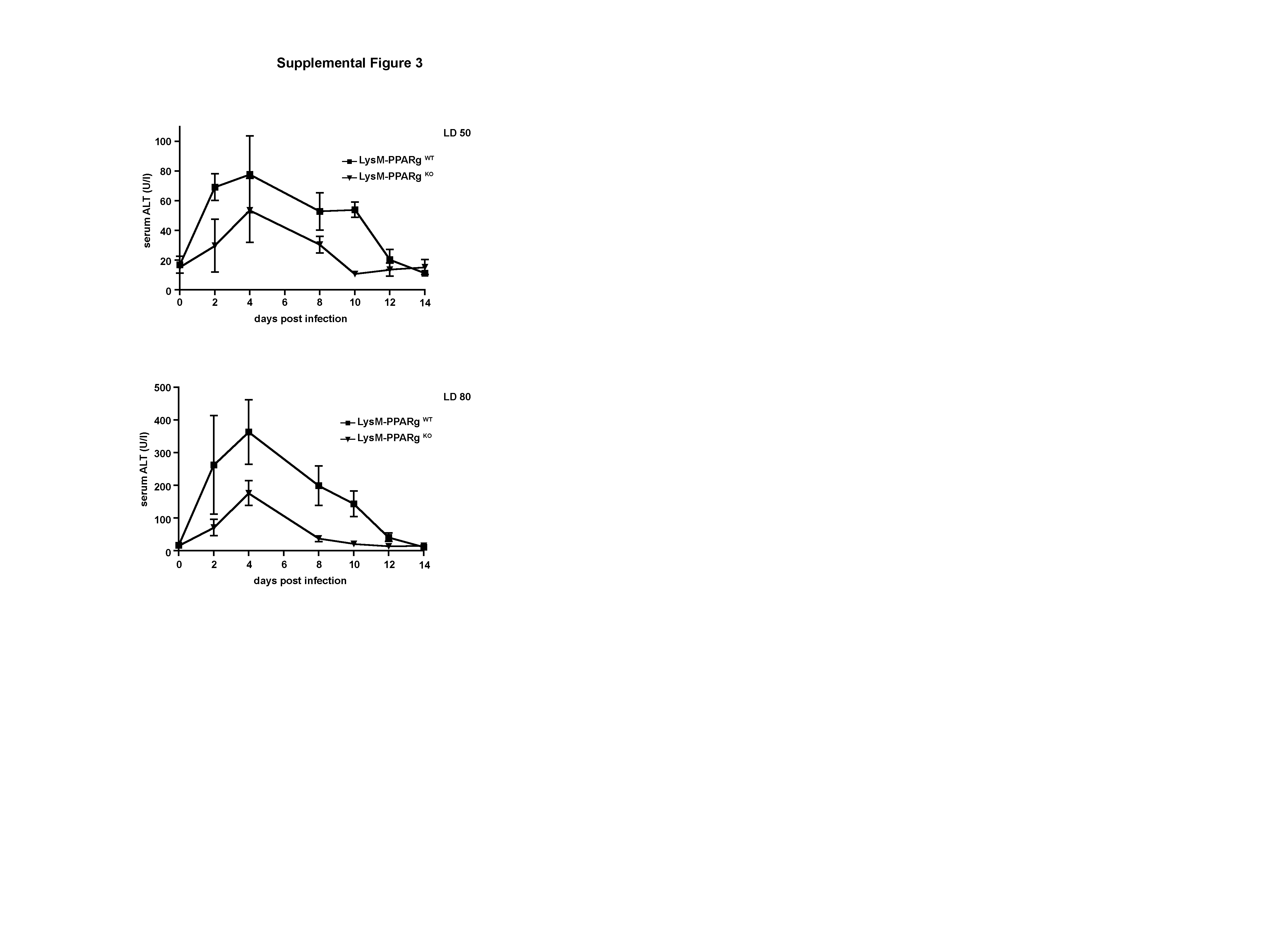

Supplement: Figure S3 — Determination of serum ALT levels. Alanine aminotransferase levels in serum were determined in mice LysM-PPARγWT and LysM-PPARγKO mice following infection with different doses of L. monocytogenes. (TIF) [file pone.0037349.s003.tif]

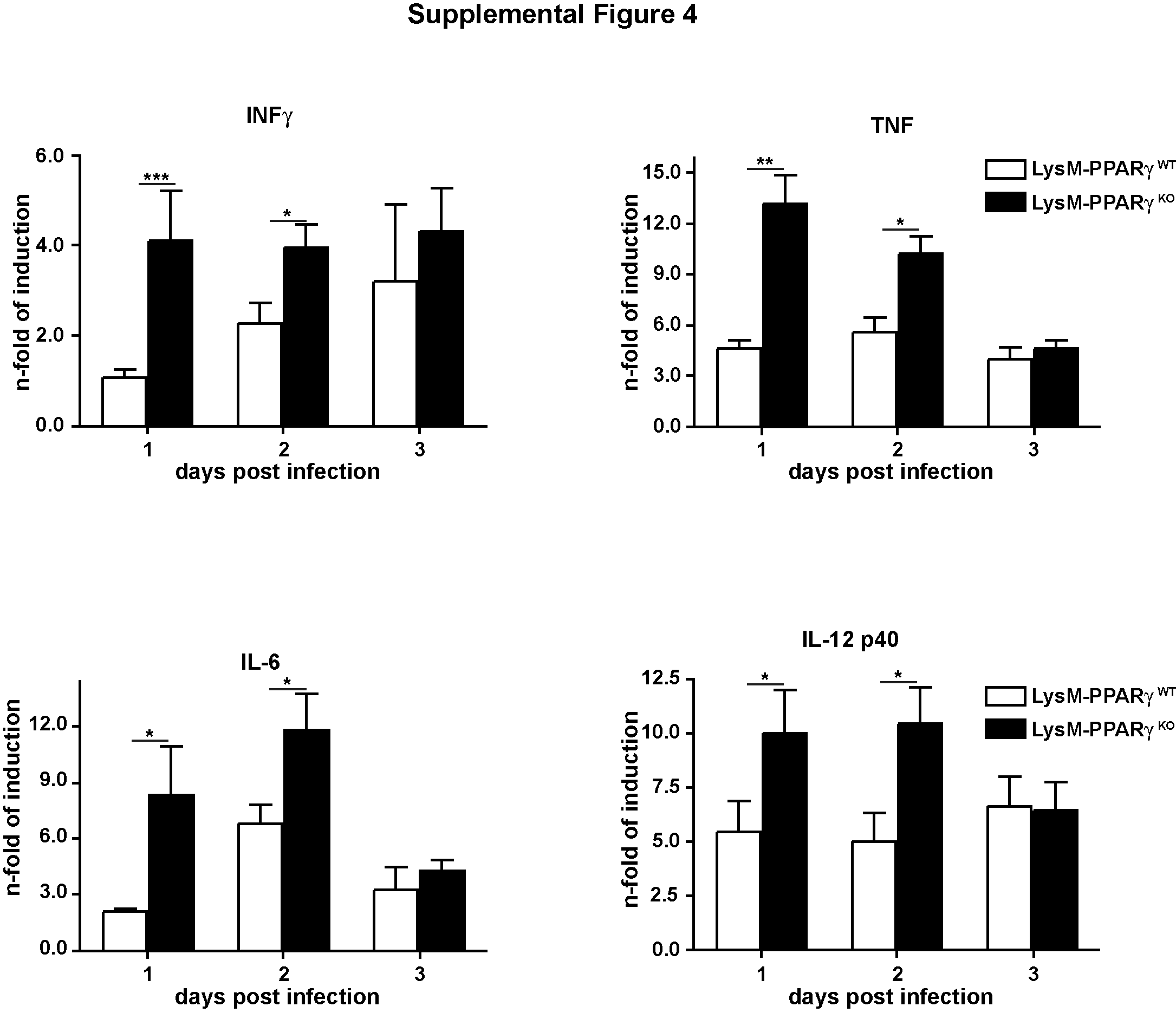

Supplement: Figure S4 — Enhanced expression of inflammatory cytokies in PPARγKO macrophages during Listeria infection in vivo. LysM-PPARγWT and LysM-PPARγKO mice were infected with 2×10^4^ CFU of L. monocytogenes. At indicated time points peritoneal macrophages were isolated and the expression of IFNγ, TNFα, IL-6 and IL-12 was assessed by qRT-PCR. (TIF) [file pone.0037349.s004.tif]

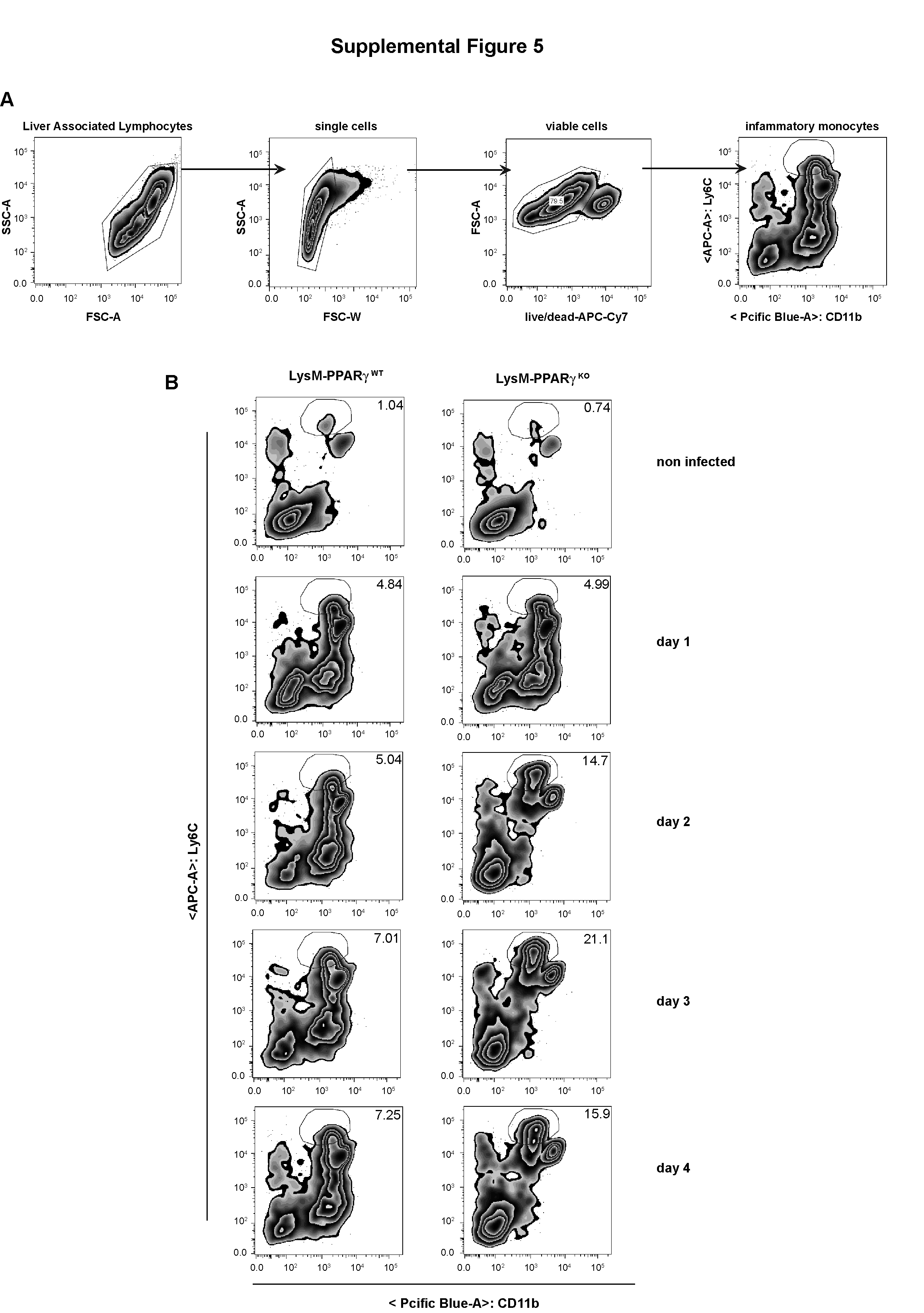

Supplement: Figure S5 — Gating strategy and representative flow cytometric data for increase of inflammatory monocytes. LysM-PPARγWT and LysM-PPARγKO mice were infected i.p. with Listeria (2×10^4^ CFU). (A) Gating strategy for identifying viable inflammatory monocytes. (B) At indicated time points the frequency of CD11b+ Ly6Chigh monocytes within viable cells isolated from the liver were determined by FACS analysis. Quantification of inflammatory monocyte cell numbers was done using fluorochrome-labeled microbeads (CountBright absolute counting beads, life technologies, Invitrogen). (TIF) [file pone.0037349.s005.tif]

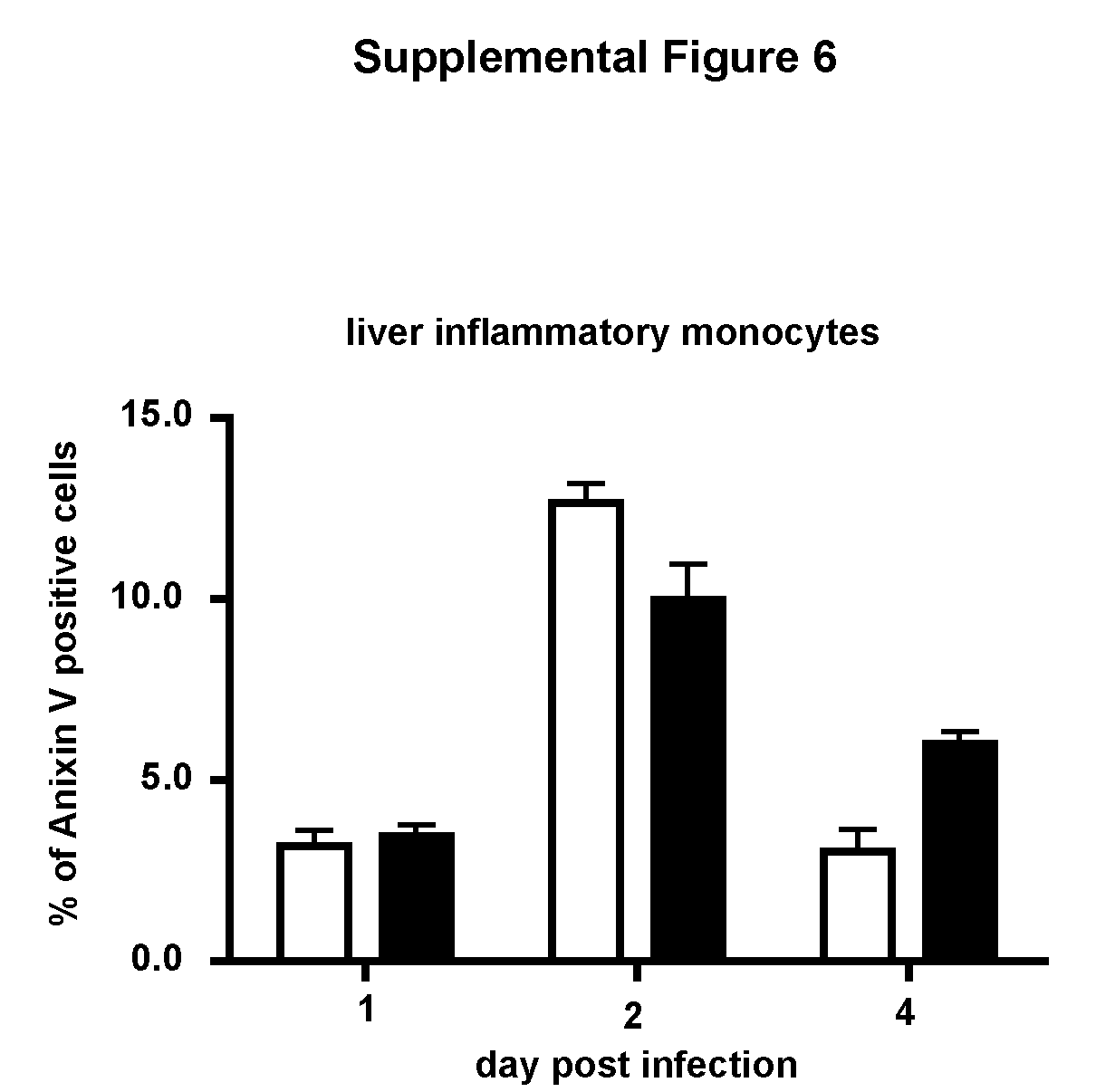

Supplement: Figure S6 — Annexin V staining of inflammatory monocytes from LysM-PPARγWT and LysM-PPARγKO mice. Detection of Annexin V levels on the surface of inflammatory monocytes isolated from the liver of Listeria-infected LysM-PPARγWT and LysM-PPARγKO mice at the indicated time points. 3 mice per group were analysed, one out of three representative experiments is shown. (TIF) [file pone.0037349.s006.tif]

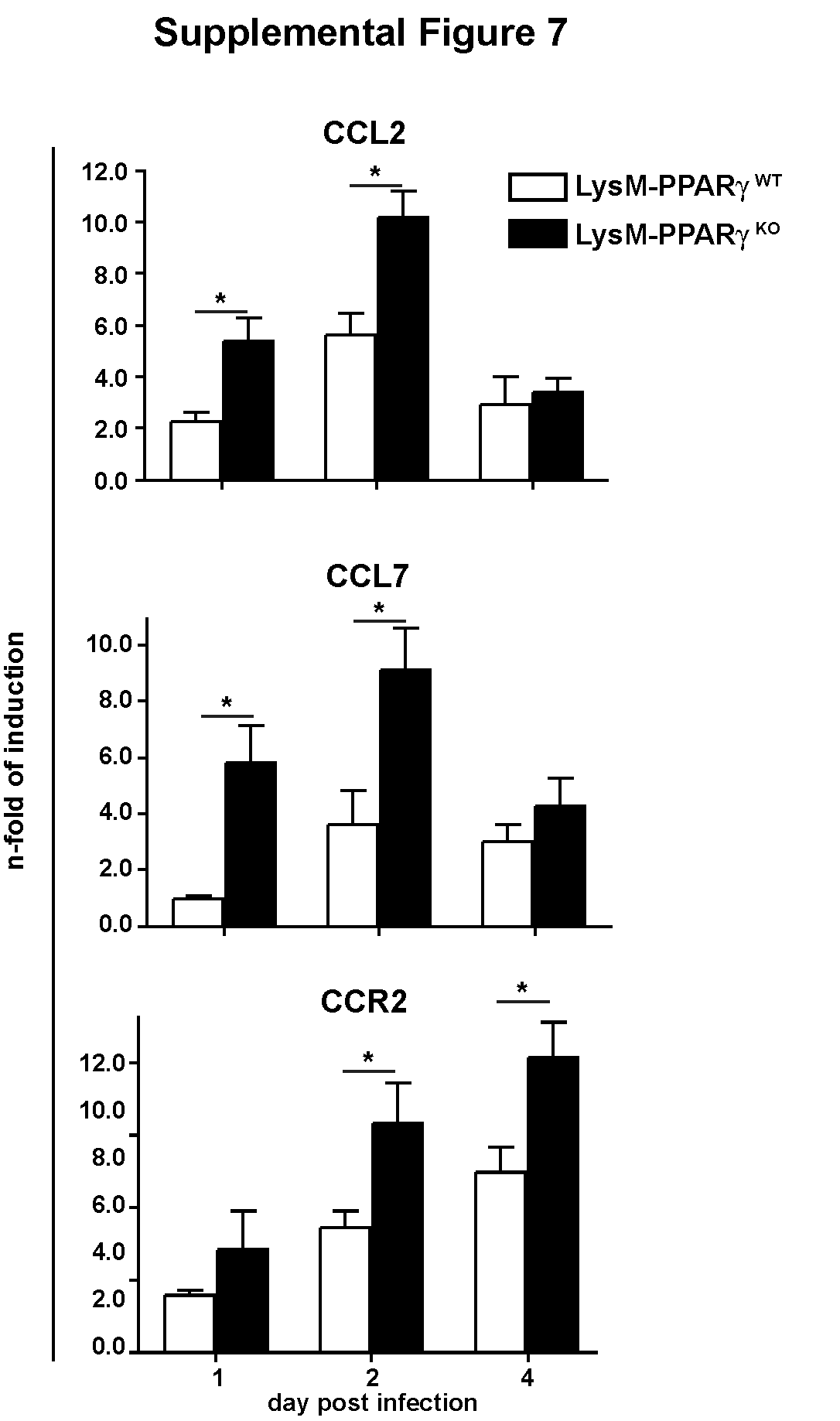

Supplement: Figure S7 — Enhanced expression of CCL2 and CCL7 and their receptor CCR2 in PPARγKO macrophages during Listeria infection in vivo. LysM-PPARγWT and LysM-PPARγKO mice were infected with 2×10^4^ CFU of Listeria. At indicated time points peritoneal macrophages were isolated and the expression of CCL2, CCL7 and CCR2 was assessed by qRT-PCR. (TIF) [file pone.0037349.s007.tif]
