# Supplementary material for: Lack of PPARγ in Myeloid Cells Confers Resistance to Listeria monocytogenes Infection
Source: PLoS One. 2012 May 21;7(5):e37349. doi: 10.1371/journal.pone.0037349 (PMC3357414; doi:10.1371/journal.pone.0037349)
Supplement: Table S1 — List of arrays performed. (DOCX) [file pone.0037349.s008.docx]

| Array | Condition |
| --- | --- |
| 5376074042_E | Macrophages infected with wild type L.m. |
| 5376074042_G | Macrophages infected with wild type L.m. |
| 5376074042_J | Macrophages infected with wild type L.m. |
| 4648883071_A | uninfected Macrophages |
| 4648883071_D | uninfected Macrophages |
| 4958478033_L | uninfected Macrophages |
| 4958478044_J | uninfected Macrophages |
| 5342595020_B | uninfected Macrophages |
| 5342595020_H | uninfected Macrophages |
| 5342595020_L | uninfected Macrophages |
|  |  |
